# Supplementary material for: Observing time-dependent energy level renormalisation in an ultrastrongly coupled open system
Source: Nat Commun. 2025 Mar 13;16:2502. doi: 10.1038/s41467-025-57840-4 (PMC11906871; doi:10.1038/s41467-025-57840-4)
Supplement: Supplementary file 1 — Supplementary Information [file 41467_2025_57840_MOESM1_ESM.pdf]

Supplementary Information for Observing Time-Dependent Energy Level Renormalisation in an Ultrastrongly Coupled Open System

Alessandra Colla
Institute of Physics, University of Freiburg, Hermann-Herder-Straße 3, D-79104 Freiburg, Germany and Dipartimento di Fisica Aldo Pontremoli, Università degli Studi di Milano, Via Celoria 16, I-20133 Milan, Italy

Florian Hasse and Deviprasath Palani
Institute of Physics, University of Freiburg, Hermann-Herder-Straße 3, D-79104 Freiburg, Germany

Tobias Schaetz and Heinz-Peter Breuer
Institute of Physics, University of Freiburg, Hermann-Herder-Straße 3, D-79104 Freiburg, Germany and EUCOR Centre for Quantum Science and Quantum Computing, University of Freiburg, Hermann-Herder-Straße 3, D-79104 Freiburg, Germany

Ulrich Warring
Institute of Physics, University of Freiburg, Hermann-Herder-Straße 3, D-79104 Freiburg, Germany

These authors contributed equally: Alessandra Colla, Florian Hasse
Corresponding author Ulrich Warring ulrich.warring@physik.uni-freiburg.de
(Dated: February 3, 2025)

COMPARISON OF MODEL DESCRIPTIONS

This section compares two seminal models in quantum mechanics: the Quantum-Rabi (QR) model, its Jaynes-Cummings (JC) approximation, and the Trapped-Ion (TI) model. The QR model, integral to quantum optics, describes interactions between a two-level atom and a single electromagnetic field mode. In its simplest form with rotating wave approximation (RWA) it is typically called the JC model. The TI model, instrumental in experimental quantum information, simulation, and metrology applications, focuses on well-isolated atomic ions trapped in electromagnetic traps. Despite their distinct origins, both models, when the TI model is viewed under Lamb-Dicke (LD) and RWA, converge for certain parameter regimes in their descriptions of light-matter interactions and experimentally accessible analogues of it: Generally, the trapped-ion platform can be tuned to match the JC dynamics and study analogue features experimentally while using analytical JC model predictions. See Supplementary Table **1** for an overview of relevant parameters and approximations. We can study and quantify the similarity between the two models using the trace distance of the two-level subsystem using a numerical approach implemented in QuTiP.

For a comprehensive comparison, we examine the time evolution of $\langle\sigma_x\rangle$, $\langle\sigma_y\rangle$, and $\langle\sigma_z\rangle$ in both models (see Supplementary Figure **1**). The best match between the models is given by taking the coupling parameter in the JC model as $g = \eta\Omega_R/2$ and the two-level system level spacing as $\omega = ((\omega^*)^2 + \Omega_R^2)^{1/2}$, cf. see Supplementary Table **1**. This is necessary to account for the carrier term, which is present in the TI case, resulting from the first order expansion of the TI coupling in the LD approximation. This term also induces a basis rotation on the spin system, which can be typically neglected for $\omega \gg \Omega_R$. The differences in dynamics found in Supplementary Figure **1** highlight the impact of fast-rotating and non-linear coupling terms in the TI model.

This analysis illustrates the nuanced differences in dynamics between the models, with the TI model's rapid rotations and nonlinearities contrasting the simpler JC dynamics. Repeated measurements are necessary to discern these subtle effects amidst quantum projection noise (QPN) [4, 5] and technical noise contributions. We illustrate the effects of the fundamental QPN for our type of measurement in Supplementary Figure **2** and indicate the required efforts and stability of the system S.

EMERGENT HAMILTONIAN FOR THE JC MODEL

The exact master equation for the two-level system in the JC model can be derived following [6] for initial environmental states that commute with the number operator. The effective Hamiltonian and thus the time-dependent shift can then be evaluated exactly depending on the initial state of the mode. For example, for a thermal initial environmental state $\rho_E(0) = e^{-\beta\omega_m a^\dagger a}/\mathrm{Tr}\{e^{-\beta\omega_m a^\dagger a}\}$, we obtain the following time-dependent frequency shift

$$\delta\tilde{\omega}(t) = -\mathrm{Im}\left\{\frac{\dot{\gamma}(t)}{\gamma(t)}\right\} , \tag{S1}$$

Supplementary Table 1. **Comparison of the Quantum-Rabi model – approximated by the Jaynes-Cummings (JC) model – and the Trapped-Ion model – approximated in the so-called red sideband and Lamb-Dicke (LD) regime.** The parameters are defined as follows: ω (part of the system S) and ω^* (System S^*) are the two-level and effectively bare, laser-dressed two-level (pseudo-spin) frequencies respectively, ω_m (part of system/environment E) represents the harmonic oscillator (photon/phonon mode) frequency, g is the coupling strength of the QR model, $\Omega_{\mathrm{R}}/(2\,\pi)$ is the Rabi frequency of the spin-mode coupling in the TI model, a and $a^\dagger$ are the annihilation and creation operators of the modes, σ_- and σ_+ are the lowering and raising operators, σ_z are the Pauli z-operators, and η is the LD parameter. The non-linear TI coupling operator $C(\eta, a, a^\dagger) = \exp\left[\mathrm{i}\eta(a^\dagger + a)\right]$ simplifies to $C_{\mathrm{LD}}(\eta, a, a^\dagger) = 1 + \mathrm{i}\eta(a^\dagger + a)$ under the Lamb-Dicke approximation (LDA) for $\eta \ll 1$. For details on derivations and explanations, see Refs.[1–3]. In the case of LDA and RWA, both approximated interaction descriptions converge, and we identify $\omega = ((\omega^*)^2 + \Omega_R^2)^{1/2}$, and $g = \eta\Omega_{\mathrm{R}}/2$.

	Quantum-Rabi Model	Trapped-Ion Model
System S/S^* (atom/spin)	$\hbar\omega\sigma_z/2$	$\hbar\omega^*\sigma_z/2$
Environment E (cavity/phonon mode)	$\hbar\omega_m a^\dagger a$	$\hbar\omega_m a^\dagger a$
Full S/S^*-E interactions	$\hbar g(a^\dagger + a)(\sigma_- + \sigma_+)$	$\hbar\Omega_{\mathrm{R}}/2\,[C(\eta, a, a^\dagger)^\dagger\sigma_-$ $+C(\eta, a, a^\dagger)\sigma_+]$
Approx. interactions [a]	$\hbar g(a^\dagger\sigma_- + a\sigma_+)$	$\hbar\eta\Omega_{\mathrm{R}}/2(a^\dagger\sigma_- + a\sigma_+)$

[a] In the case of the TI model, the approximation is called the red sideband regime [1, 2]

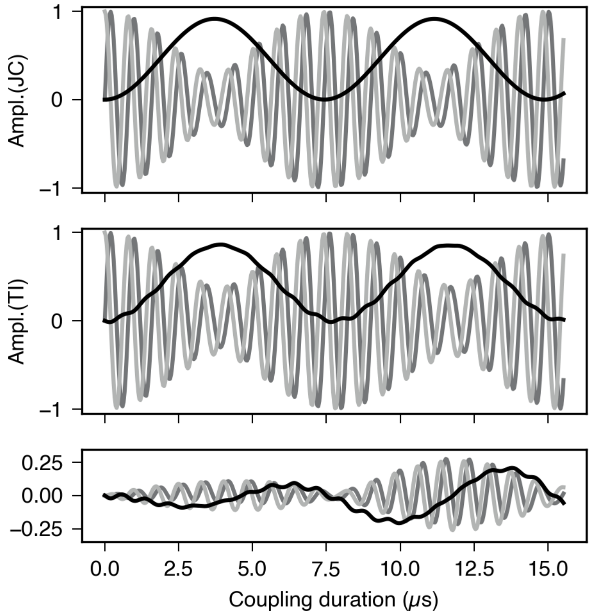

Supplementary Figure 1. **Numerical comparison between the JC and TI models.** The numerical evolution of expectation values following the JC model (top) and TI model (middle) are shown for different spin components as a function of coupling duration: $\langle\sigma_x\rangle$ in light grey, $\langle\sigma_y\rangle$ in grey, and $\langle\sigma_z\rangle$ in black. Parameters are matched in the numerical simulation except for the modified ω in JC with respect to the TI ω^*. Differences (bottom graph) in dynamics underscore the effects of fast-rotating and non-linear terms in the TI model.

where

$$\gamma(t) = \left(1 - e^{-\beta\omega_m}\right)\sum_{n=0}^{\infty} c(n,t)c(n+1,t)e^{-\beta\omega_m n}\ . \quad \text{(S2)}$$

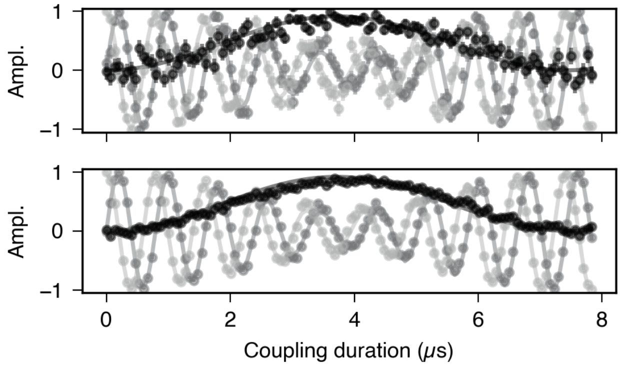

Supplementary Figure 2. **Numerical comparison of the Jaynes-Cummings model ($H_{\mathbf{JC}}^{\mathbf{RWA}}$) and the full Trapped Ion model ($H_{\mathbf{TI}}$) with fundamental quantum projection noise (QPN).** We simulate the effect of QPN with 50 and 500 repetitions per data point and compare in total 100 data points with estimates from $H_{\mathrm{JC}}^{\mathrm{RWA}}$ (solid lines). Expectation values $\langle\sigma_x\rangle$, $\langle\sigma_y\rangle$, and $\langle\sigma_z\rangle$ are displayed in black, grey, and light grey, respectively. Collecting these data sets approximates 5 and 30 minutes of measurement time. Error bars represent the standard error of the mean.

Here we have introduced the coefficients

$$c\,(n,t) = e^{-i\Delta t/2}\left[\cos\left(\Omega_n\frac{t}{2}\right) + i\Delta\frac{\sin\left(\Omega_n\frac{t}{2}\right)}{\Omega_n}\right]\ , \quad \text{(S3)}$$

with $\Omega_n = \sqrt{\Delta^2 + 4g^2 n}$. Note that the shift, Eq. (S1), depends nontrivially on the initial inverse temperature β, on the detuning Δ, and on the coupling strength g.

If the mode is initially in the vacuum, $\rho_E(0) = |0\rangle\langle 0|$, the renormalised frequency shift with respect to the bare frequency has the analytical expression

$$\delta\tilde{\omega}(t) = -\frac{2g^2}{\Delta}\frac{1}{1 + \frac{\Omega_1^2}{\Delta^2}\cot^2\left(\frac{\Omega_1}{2}t\right)}\ , \quad \text{(S4)}$$

where we have defined the Rabi frequency $\Omega_1 := \sqrt{\Delta^2 + 4g^2}$. Thus, the effective Hamiltonian shows a time-dependent driving of the spin frequency even when the mode is in the ground state. It is periodic (with period $T(\Delta) = 2\pi/\Omega_1$) and with a sign dependent on the sign of the detuning Δ.

The average renormalisation in the case of the vacuum is given by the average of the time-dependent shift over one period. We obtain:

$$\begin{aligned}\langle \delta\tilde{\omega}(t)\rangle_T &= -\frac{\Omega_1}{2\pi}\frac{2g^2}{\Delta}\int_0^{2\pi/\Omega_1} dt \frac{1}{1+\frac{\Omega_1^2}{\Delta^2}\cot^2\left(\frac{\Omega_1}{2}t\right)} \\ &= -\frac{2g^2\mathrm{sign}(\Delta)}{\Omega_1 + |\Delta|} \,. \end{aligned} \tag{S5}$$

In the dispersive limit of large detuning — namely, $|\Delta| \gg g$ — the average shift becomes

$$\langle \delta\tilde{\omega}(t)\rangle_T \xrightarrow{|\Delta|\gg g} -\frac{g^2}{\Delta} \,, \tag{S6}$$

which is the known Lamb shift for the JC model [7]

Furthermore, one can check that the time-averaged renormalised frequency in the vacuum case is given exactly by one of the two dressed state energies of the JC Hamiltonian in the one excitation manifold, either the lower or the higher energy depending on the detuning. Indeed, it holds

$$\omega + \langle \delta\tilde{\omega}(t)\rangle_T = E_- \theta(\Delta) + E_+[1-\theta(\Delta)] \,, \tag{S7}$$

with

$$E_\pm = (\omega_m + \omega \pm \Omega_1)/2 \,, \tag{S8}$$

the dressed energies associated with the eigenstates of the JC Hamiltonian.

SUPPLEMENTARY REFERENCES

[1] D. J. Wineland, C. Monroe, W. M. Itano, D. Leibfried, B. E. King, and D. M. Meekhof, "Experimental Issues in Coherent Quantum-State Manipulation of Trapped Atomic Ions," J. Res. Natl. Inst. Stand. Technol. **103**, 259–328 (1998).

[2] D. Leibfried, R. Blatt, C. Monroe, and D. Wineland, "Quantum dynamics of single trapped ions," Rev. Mod. Phys. **75**, 281–324 (2003).

[3] G. Clos, D. Porras, U. Warring, and T. Schaetz, "Time-Resolved Observation of Thermalization in an Isolated Quantum System," Phys. Rev. Lett. **117**, 170401 (2016).

[4] W. M. Itano, J. C. Bergquist, J. J. Bollinger, J. M. Gilligan, D. J. Heinzen, F. L. Moore, M. G. Raizen, and D. J. Wineland, "Quantum projection noise: Population fluctuations in two-level systems," Phys. Rev. A **47**, 3554–3570 (1993).

[5] M. Wittemer, G. Clos, H.-P. Breuer, U. Warring, and T. Schaetz, "Measurement of quantum memory effects and its fundamental limitations," Phys. Rev. A **97**, 020102(R) (2018).

[6] A. Smirne and B. Vacchini, "Nakajima-Zwanzig versus time-convolutionless master equation for the non-Markovian dynamics of a two-level system," Phys. Rev. A **82**, 022110 (2010).

[7] M. Brune, P. Nussenzveig, F. Schmidt-Kaler, F. Bernardot, A. Maali, J. M. Raimond, and S. Haroche, "From Lamb shift to light shifts: Vacuum and subphoton cavity fields measured by atomic phase sensitive detectiont," Phys. Rev. Lett. **72**, 3339–3342 (1994).
